# Minocycline for sporadic and hereditary cerebral amyloid angiopathy (BATMAN): study protocol for a placebo-controlled randomized double-blind trial

**DOI:** 10.1186/s13063-023-07371-4

**Published:** 2023-06-05

**Authors:** S. Voigt, E. A. Koemans, I. Rasing, E. S. van Etten, G. M. Terwindt, F. Baas, K. Kaushik, A. C. G. M. van Es, M. A. van Buchem, M. J. P. van Osch, M. A. A. van Walderveen, C. J. M. Klijn, M. M. Verbeek, L. van der Weerd, M. J. H. Wermer

**Affiliations:** 1 Department of Neurology, Leiden University Medical Center, Albinusdreef 2, 2300 RC Leiden, The Netherlands; 2 Department of Radiology, Leiden University Medical Center, Albinusdreef 2, 2300 RC Leiden, The Netherlands; 3 Department of Clinical Genetics, Leiden University Medical Center, Leiden, The Netherlands; 4 Department of Neurology, Donders Institute for Brain, Cognition and Behaviour, Radboud University Medical Center, Nijmegen, The Netherlands

**Keywords:** Cerebral amyloid angiopathy, Minocycline, Dutch-type CAA, Cerebrospinal fluid, 7T MRI, Randomized controlled trial

## Abstract

**Background:**

Cerebral amyloid angiopathy (CAA) is a disease caused by the accumulation of the amyloid-beta protein and is a major cause of intracerebral hemorrhage (ICH) and vascular dementia in the elderly. The presence of the amyloid-beta protein in the vessel wall may induce a chronic state of cerebral inflammation by activating astrocytes, microglia, and pro-inflammatory substances. Minocycline, an antibiotic of the tetracycline family, is known to modulate inflammation, gelatinase activity, and angiogenesis. These processes are suggested to be key mechanisms in CAA pathology. Our aim is to show the target engagement of minocycline and investigate in a double-blind placebo-controlled randomized clinical trial whether treatment with minocycline for 3 months can decrease markers of neuroinflammation and of the gelatinase pathway in cerebrospinal fluid (CSF) in CAA patients.

**Methods:**

The BATMAN study population consists of 60 persons: 30 persons with hereditary Dutch type CAA (D-CAA) and 30 persons with sporadic CAA. They will be randomized for either placebo or minocycline (15 sporadic CAA/15 D-CAA minocycline, 15 sporadic CAA/15 D-CAA placebo). At *t* = 0 and *t* = 3 months, we will collect CSF and blood samples, perform a 7-T MRI, and collect demographic characteristics.

**Discussion:**

The results of this proof-of-principle study will be used to assess the potential of target engagement of minocycline for CAA. Therefore, our primary outcome measures are markers of neuroinflammation (IL-6, MCP-1, and IBA-1) and of the gelatinase pathway (MMP2/9 and VEGF) in CSF. Secondly, we will look at the progression of hemorrhagic markers on 7-T MRI before and after treatment and investigate serum biomarkers.

**Trial registration:**

ClinicalTrials.gov NCT05680389. Registered on January 11, 2023

**Supplementary Information:**

The online version contains supplementary material available at 10.1186/s13063-023-07371-4.

## Background

Cerebral amyloid angiopathy (CAA) is a disease caused by the accumulation of the protein amyloid-beta in the leptomeningeal arteries, cortical arterioles, and capillaries of the brain. It is one of the major causes of intracerebral hemorrhage (ICH) and vascular dementia in the elderly. Approximately 60% of all lobar ICHs are CAA related [1].

Most CAA cases are sporadic, but a few familial forms exist. Several families suffer from hereditary Dutch-type cerebral amyloid angiopathy (D-CAA), an autosomal dominant familial form of CAA. D-CAA is caused by a genetic mutation in the amyloid-beta precursor-protein-gene (APP) on chromosome 21 [2]. D-CAA, like sporadic-CAA, is characterized by recurrent ICH, cognitive decline, and dementia, although its disease course is more aggressive [3].

Currently, there is no treatment for D-CAA or sporadic CAA. So far, only one phase 2 trial with a monoclonal antibody against amyloid-beta has been performed in sporadic CAA, but treatment worsened cerebrovascular reactivity on MRI [4]. Other amyloid-targeting therapies (RNA interference therapies, amyloid antibodies, and antisense-oligonucleotide treatment) hold promise but are still in the preclinical phase.

The presence of amyloid-beta in the vessel wall induces a chronic state of cerebral inflammation by reactive astrocytes, microglia, and pro-inflammatory substances [5, 6]. Previous neuropathological studies in patients with D-CAA have shown that cerebral inflammation is abundant in brain tissue. In addition, gelatinase activity was increased (metalloproteinase [MMP]-2 and MMP-9) leading to extracellular matrix remodeling and reduced blood-brain-barrier integrity [7, 8]. MMP-2 and MMP-9 activities are related to microbleed development in CAA mouse models [9, 10]. Furthermore, D-CAA patients show upregulated VEGF expression and angiogenesis pathways [8]. Moreover, in both sporadic and hereditary CAA, subacute neurological symptoms such as confusion and seizures may occur with signs of CAA-related inflammation on MRI (CAA-ri) [11].

Minocycline is an antibiotic of the tetracycline family and is known to modulate inflammation, gelatinase activity, and angiogenesis. These processes are suggested to be key mechanisms in CAA pathology. Minocycline is an EMA-approved antibiotic and is freely available as it is no longer protected by a patent. It is unique in its low costs, limited side effects, and easy availability. Pre-clinical studies with minocycline in different CAA animal models showed promising results [12–15]. Although minocycline did not reduce the amount of vascular amyloid-beta deposition, the drug prevented ICH occurrence and improved behavioral outcomes in mice. Biochemical and imaging analyses showed that minocycline significantly reduced gliosis and expression of inflammatory genes and gelatinases (e.g., IL-6, IBA-1, MMPs). These studies did not include an analysis of VEGF or angiogenic activity, but VEGF is one of the most well-known targets of minocycline [16]. Based on the hypothesis that minocycline reduces inflammatory responses, we will perform a randomized clinical phase 2a trial with minocycline as a treatment for CAA.

## Methods

### Study design

**Fig. 1:**
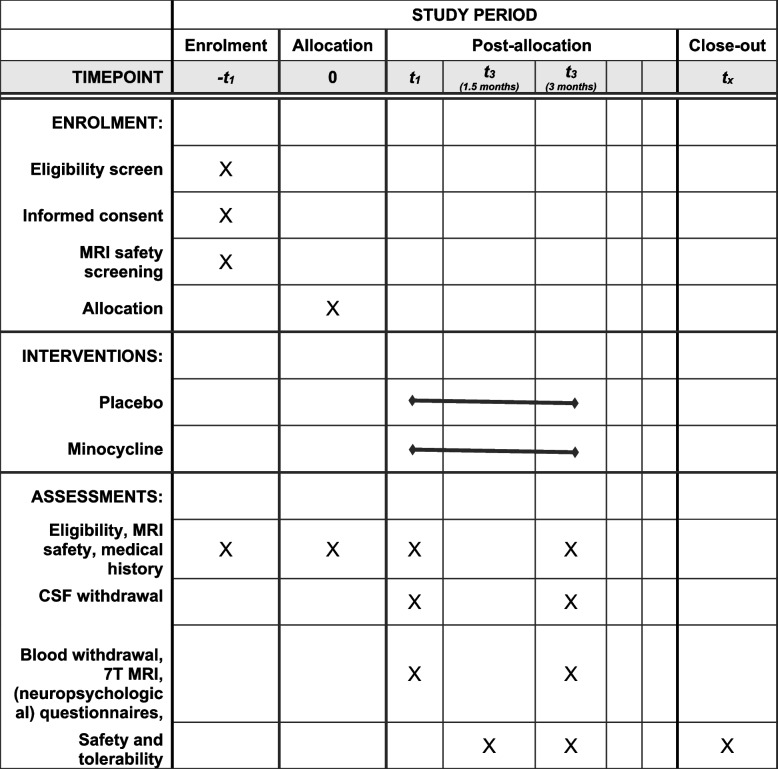
Schedule of enrollment, interventions, and assessments

This is an exploratory study. Our study design is a randomized double-blind placebo-controlled trial with minocycline treatment versus placebo for 3 months in 60 participants (30 with D-CAA and 30 with sporadic CAA). At baseline (*t* = 0), a standardized neuropsychological assessment will be performed (Fig. 1). At *t* = 1 and *t* = 3 months, we will perform the following study procedures: CSF sample collection, collection of demographics, neurological examination, blood sample collection, and 7-T MRI (Fig. 1). In-between study visits, a short questionnaire on side effects and compliance will be sent to enhance participant retention.

### Characteristics of the participants

The inclusion criteria for the participants with D-CAA are the age of 18 years and older and genetically proven D-CAA. For participants with sporadic CAA, we used the modified Boston criteria [17]. Furthermore, all participants should have no more than two ICHs (with the occurrence of the last ICH at least 1 year ago, to ensure that inflammation in the acute stage of ICH is stabilized). Also, all participants should have at least two lobar microbleeds or cortical superficial siderosis on MRI. With these criteria, we hope to include participants with moderate CAA pathology but not yet severe.

The exclusion criteria are a previous allergic reaction to minocycline, a modified Rankin Scale score of at least 3, contraindications for 7-T MRI (e.g., claustrophobia, pacemakers, and ferromagnetic implants), contraindications for lumbar puncture (e.g., compression of the spinal cord, a coagulopathy or thrombocytopenia < 100), pregnancy or breastfeeding, liver or renal failure, use of antibiotics 1 month prior of participation, systemic lupus erythematosus or other diseases known to generate inflammatory responses, and use of drugs that are contraindicated in combination with minocycline (e.g., carbamazepine).

### Randomization and treatment dose

There will be a block randomization by computer for both forms of CAA to ensure 15 participants in each group (D-CAA 15 minocycline and 15 placebo, sporadic CAA 15 minocycline and 15 placebo). Minocycline will be administered orally twice a day 100 mg (total daily dose = 200mg) for 3 months.

### Treatment allocation and blinding

The pharmacy of the LUMC will perform block randomization by computer, generate the allocation sequence, and blindly repackage the products. Everyone except the pharmacy will be blinded (trial participants, care providers, outcome assessors, and data analysts). After the termination of the study and analysis of the data, data analysts and trial participants will be unblinded.

### Primary endpoint

Our primary outcome measures are CSF biomarkers of inflammation, vessel integrity, and the gelatinase pathway with emphasis on IL-6, MCP-1, IBA-1, MMP2/9, and VEGF. CSF is collected at *t* = 0 and *t* = 3 months.

### Secondary endpoints

The secondary endpoints include the safety and tolerability of minocycline and hemorrhagic markers on 7-T MRI and serum biomarkers. These data are collected at *t* = 0 and *t* = 3 months. Furthermore, we will use the collected data to obtain information on the stability and natural variation of different CSF biomarkers and blood biomarkers in patients with CAA over time.

### Sample size calculations

This is an exploratory study, and a formal power calculation is difficult as the levels of inflammatory CSF biomarkers in D-CAA or sporadic CAA are not well known. In our D-CAA-cohorts, previous CSF analyses of amyloid-beta 40 and 42 and a biomarker panel including, e.g., VEGF, showed that patients could be distinguished from controls with group sizes as small as 15 [18]. In stroke patients, there was a high correlation between serum levels of IL-6 and MMP-9 and the occurrence of microbleeds [19, 20]. Previous animal studies using minocycline showed a 30–70% reduction in relevant biomarkers such as MMP-9 and gliosis on a tissue level [13, 15]. In our data, VEGF levels in CSF showed a twofold increase in patients compared with controls (6.5 ± 2.8 vs 3.2 ± 2.2 pg/ml). Assuming a similar effect size of 50% change after treatment (power of 0.8, alpha = 0.05), the calculated group size would be 14. As the effect may be less robust compared to the animal model, we chose a group size of 30 participants, which would yield sufficient power to detect a 30% change, based on the preliminary data. Individual subjects will be replaced after withdrawal. Outcome data for all included participants will be collected.

### Statistical analysis

We will perform descriptive statistics for the prevalence of different biomarkers in CSF (and MR imaging) and their evolution over time with disease progression.

Because minocycline targets multiple pathways (including inflammatory, vessel integrity, and gelatinase pathways) that play a role in CAA, we chose multiple CSF biomarkers to cover all three pathways. We will compare the level of inflammatory biomarkers between the treated and not treated groups with regression analyses, adjusted for age and sex. In the exploratory subgroup analyses, we will stratify for D-CAA and sporadic CAA and will use repeated measurements analyses, as well as use a single primary composite measure [21, 22]. Since this is an exploratory study, we will not correct for multiple testing for the 5 (IL-6, MCP-1, IBA-1, MMP2/9, and VEGF) co-primary endpoints.

### Ethical considerations

There is no anticipated harm and compensation for trial participation.

## Discussion

The BATMAN study investigates the effect of minocycline on CSF markers of neuroinflammation and the gelatinase pathway in patients with CAA. To the best of our knowledge, this is the first repurpose drug study in CAA.

Our choice for a dosage of 200 mg/day was based on a recent RCT of minocycline in Alzheimer’s disease, in which different dosages were used (200 mg/day and 400 mg/day) for 24 months [23]. This study reported that 400 mg was tolerated quite poorly, while 200 mg was well tolerated. The dose of 200 mg is also used in a recently started phase 2 trial on the effect of minocycline on inflammation and blood barrier leakage in patients with lacunar stroke and white matter hyperintensities [24]. The employed dosage has been shown to produce consistent anti-inflammatory effects in rheumatoid arthritis and other inflammatory disorders and is also used in recent trials for neuropsychiatric diseases [25].

Our choice for a duration of 3 months was based on the fact that in chronic skin infections (e.g., acne vulgaris), minocycline is used for a maximum of 6 months. However, the anti-inflammatory and vasoprotective actions of minocycline are expected to occur within 3 weeks [16]. Therefore, we expect that the 3-month duration provides sufficient time to detect a biomarker effect, while minimizing drop-out. Intermediate side effects will be monitored, and in case side effects occur, phasing out will be considered.

The duration of 3 months may be too short to detect an effect on microvascular damage on MRI, and this analysis is considered to be exploratory. However, ultra-high-field 7-T MRI leads to better visibility of small structures and increased signal-to-noise ratio [26]. Previous studies shown that 7 T is more sensitive for detecting microbleeds than 3 T [27–29].

When our study shows a decrease in CSF biomarkers in the minocycline group, a larger trial would be needed to assess the clinical efficacy. If a larger trial shows treatment with minocycline improves clinical outcomes in patients with CAA, by slowing down the progression of the disease or improving recovery after ICH, it would represent a unique therapeutic option for CAA.

## Trial status

The first participant was included on 2 December 2020. At the time of submission of this article, 43 participants have been enrolled in the trial. During the COVID-19 pandemic, the study was put on hold temporarily. Recruitment is estimated to be completed in December 2023.

## Supplementary Information

**Additional file 1:** **Supplementary Table 1.** BATMANR1.

## Data Availability

The principal investigator (MJHW) and the first author (SV) will have access to the final trial dataset. Any data required to support the protocol can be supplied on request. The participant information materials and informed consent form are available from the corresponding author on request. The datasets analyzed during the current study and statistical code are available from the corresponding author on reasonable request, as is the full protocol.

## Abbreviations

Aβ: Amyloid beta
APP: Amyloid precursor protein
CAA: Cerebral amyloid angiopathy
CMB: Cerebral microbleeds
CSF: Cerebrospinal fluid
cSS: Cortical superficial siderosis
D-CAA: Hereditary Dutch-type cerebral amyloid angiopathy
HCHWA-D: Hereditary cerebral hemorrhage with amyloidosis-Dutch type
ICH: Intracerebral hemorrhage
MRI: Magnetic resonance imaging
7T MRI: 7-T magnetic resonance imaging
