# Supplementary material for: Minocycline for sporadic and hereditary cerebral amyloid angiopathy (BATMAN): study protocol for a placebo-controlled randomized double-blind trial
Source: Trials. 2023 Jun 5;24:378. doi: 10.1186/s13063-023-07371-4 (PMC10241553; doi:10.1186/s13063-023-07371-4)
Supplement: Supplementary file 1 — Additional file 1: Supplementary Table 1. BATMANR1. [file 13063_2023_7371_MOESM1_ESM.docx]

| **Data category** | **Information** |
| --- | --- |
| Primary registry and trial identifying number | ClinicalTrials.gov NCT05680389 |
| Date of registration in primary registry | 11^th^ of January, 2023 |
| Secondary identifying numbers | NL71513.058.20 (MREC, LUMC) |
| Source(s) of monetary or material support | The Netherlands Organization for Health Research and Development (ZonMw/Hersenstichting) [project number DR-2019-00299] |
| Primary sponsor(s) | Leiden University Medical Center |
| Secondary sponsor(s) | NA |
| Contact for public queries | Sabine Voigt, s.voigt@lumc.nl |
| Contact for scientific queries | Sabine Voigt, s.voigt@lumc.nl |
| Public title | BATMAN |
| Scientific title | Minocycline for sporadic and hereditary cerebral amyloid angiopathy (BATMAN): study protocol for a placebo-controlled randomized double-blind trial |
| Protocol date and version | Version 13, 26^th^ of August 2022 |
| Countries of recruitment | The Netherlands |
| Health condition(s) or problem(s) studied | Cerebral Amyloid Angiopathy |
| Intervention(s) | Active comparator: minocycline  Placebo comparator: placebo |
| Key inclusion and exclusion criteria | Inclusion criteria for the participants with D-CAA are age of 18 years and older and genetically proven D-CAA. For participants with sporadic CAA, we used the modified Boston criteria. Furthermore, all participants should have not more than two ICHs (with occurrence of last ICH at least one year ago, to ensure that inflammation in the acute stage of ICH is stabilized). Also, all participants should have at least two lobar microbleeds or cortical superficial siderosis on MRI. With these criteria we hope to include participants with moderate CAA pathology but not yet severe. Exclusion criteria are a previous allergic reaction to minocycline, a modified Rankin Scale score of at least 3, contraindications for 7T MRI (e.g. claustrophobia, pacemakers and ferromagnetic implants), contraindications for lumbar puncture (e.g. compression of the spinal cord, a coagulopathy or thrombocytopenia <100), pregnancy or breast feeding, liver or renal failure, use of antibiotics one month prior of participation, systemic lupus erythematosus or other diseases known to generate inflammatory responses and use of drugs that are contraindicated in combination with minocycline (e.g. carbamazepine). |
| Study type | Randomized double-blind placebo-controlled trial |
| Date of first enrolment | 2^nd^ of December 2020 |
| Target sample size | 60 |
| Recruitment status | Ongoing |
| Primary outcome(s) | CSF biomarkers of inflammation, vessel integrity and the gelatinase pathway with emphasis on IL-6, MCP-1, IBA-1, MMP2/9, and VEGF. |
| Key secondary outcomes | Safety and tolerability of minocycline, as well as hemorrhagic markers on 7T MRI and serum biomarkers. Furthermore we will use the collected data to obtain information on the stability and natural variation of different CSF biomarkers and blood biomarkers in patients with CAA over time. |
